# Ventilator-Assisted Inspiratory and Expiratory Breath-Hold Thoracic Computed Tomographic Scans Can Detect Dynamic and Static Airway Collapse in Dogs with Limited Agreement with Tracheobronchoscopy

**DOI:** 10.3390/ani12223091

**Published:** 2022-11-10

**Authors:** Alice Levy, Carol Reinero, Isabelle Masseau

**Affiliations:** 1 Department of Sciences Cliniques, Faculté de Médecine Vétérinaire, Université de Montréal, St-Hyacinthe, QC J2S 2M2, Canada; 2 Department of Veterinary Medicine and Surgery, College of Veterinary Medicine, Columbia, MI 65211, USA

**Keywords:** bronchomalacia, lobar bronchial collapse, airway caliber, airway circularity, airway cross-sectional area

## Abstract

**Simple Summary:**

Airway collapse consists of the partial/complete narrowing of an airway with subsequent restriction to the airflow. It can occur anywhere along the trachea or bronchial tree in dogs and may be present throughout the respiratory cycle (i.e., static collapse) or it may be associated with a specific phase of respiration (e.g., expiration), hence “dynamic collapse”. In dogs, the clinical signs of an airway collapse may overlap those of other respiratory diseases. This study aimed to determine whether ventilator-assisted computed tomography (CT) with images acquired at inspiration and expiration would detect static and dynamic airway collapse in dogs with spontaneous respiratory disease and to compare the CT results with those obtained with tracheobronchoscopy, a modality which is commonly used to assess the airways in anesthetized dogs. The study found that the variation in the size of the airway was more profound for the trachea, the right mainstem bronchus and the right middle lobar bronchus in dogs with an airway collapse. Static collapse was only seen in the trachea of dogs with an airway collapse. The agreement between the CT and tracheobronchoscopy results was slight to moderate. The study shows that inspiratory and expiratory CT scans can detect static and dynamic airway collapse with it having limited agreement with the tracheobronchoscopy results. Scoring systems that are tailored to the clinical manifestations of function impairments may improve the comparisons in the future.

**Abstract:**

Airway collapse (AC) in dogs includes a tracheal collapse, mainstem and lobar bronchial collapse, and bronchomalacia (i.e., segmental/subsegmental bronchial collapse). The clinical presentation of AC may overlap with non-collapsible airway disease (NCAD) or another non-lower airway respiratory disease (NLARD). This study determined whether paired inspiratory (I)/expiratory (E)-breath-hold computed tomography (I/E-BH CT) can detect a static and dynamic AC in dogs with spontaneous respiratory disease and it compared the CT-derived metrics of the AC to the tracheobronchoscopy metrics. The CT-acquired I and E diameter and cross-sectional area (CSA) for the trachea, mainstem and lobar bronchi in dogs with an AC (*n* = 16), NCAD (16), and NLARD (19) served for a dynamic percent of the airway narrowing (%AN) calculation. A scoring system assessed the bronchomalacia. The circularity was calculated for each airway. The results were compared to the tracheobronchoscopy collapse grading. In the dogs with an AC, the %AN was larger for the trachea, right mainstem bronchus and right middle lobar bronchus when they were compared to the dogs with NCAD and NLARD. Flattening was only identified for the trachea of the AC dogs. The agreement between the CT and tracheobronchoscopy scores was 20% from trachea to the lobar bronchi and 47% for the segmental/subsegmental bronchi. Paired I/E-BH CT can detect static and dynamic AC with limited agreement with the tracheobronchoscopy metrics. Independent scoring systems that are tailored to the clinical manifestations of functional impairments are needed.

## 1. Introduction

Airway collapse (AC) in dogs can be subdivided anatomically to include tracheal collapse, mainstem bronchial collapse, lobar bronchial collapse and bronchomalacia. A reduced airway caliber leads to airflow obstruction with variable clinical signs depending on the location, extent and severity of the tracheal collapse, mainstem bronchial collapse, lobar bronchial collapse and bronchomalacia. Tracheal collapse and mainstem bronchial collapse affect the airways high in the tracheobronchial tree, having the potential to broadly affect all of the downstream gas exchange units. Bronchomalacia, defined as collapse of segmental/subsegmental airways [1], tends to be diffuse and may also have a marked impact on lung function. While tracheal and mainstem bronchial collapse is prevalent in middle-aged to old dogs weighing less than 10 kg [2,3,4], bronchomalacia, which is defined herein, affects dogs of all ages and weights [3]. In contrast, lobar bronchial collapse is frequently anatomically limited (e.g., only affecting one or few lobar bronchi) or has a minimal reduction in the airway caliber and contribution to the clinical signs and functional impairment. In brachycephalic breeds, bronchial collapse is common and it is speculated to be located to the secondary to upper respiratory tract obstruction [5]. The clinical relevance of a bronchial collapse, especially when it is affecting a few lobar bronchi and when it is mild, and without a concurrent tracheal or a mainstem bronchial collapse or bronchomalacia remains unclear [1].

Airway collapse can be static or dynamic, with the former lacking the change in the luminal diameter with respiration [6]. With a static collapse, the airway is often described as being deformed [7] or having a fixed W shape with respiration [8]. Circularity assesses the shape of an airway in the cross-section [9]. A collapsed/deformed airway loses its circularity. In dogs with a dynamic AC, airway caliber changes are affected by the phase of respiration, the depth of respiration and the intra- or extrathoracic location of it [8]. The diagnosis of AC can be made via a combination of radiography, fluoroscopy, computed tomography (CT) and tracheobronchoscopy [8,10,11]. The imaging modalities should be optimized to demonstrate the AC. For example, an intrathoracic collapse will be highlighted on the images that are captured during expiration when it is most severe [12]. Computed tomography is increasingly used in humans to assess dynamic AC [13,14,15]. In this instance, children > 5 years of age are instructed to first hold their breath for 5–7 s to capture the images while the lungs are completely full (end-inspiratory phase) and then, to empty their lungs and hold for another 5 to 7 s for the end-expiratory phase [16]. Infants or young children who are unable to follow the breathing instructions require intubation and controlled ventilation during which end-inspiratory and end-expiratory images are acquired while applying and withholding positive pressure ventilation, respectively [17]. Computed tomography that requires manual or mechanical ventilation with the full(end)-inspiratory/end-expiratory breath-hold (I/E-BH) technique offers the ability of capturing images with the control of the respiratory phase in dogs. Moreover, it enables the measurement and comparison of multiple locations in the tracheobronchial tree at full inspiration and expiration [11,18]. Tracheobronchoscopy has been considered to be the criterion standard for the diagnosis of AC. However, the access to multiple airways may be limited due to the logistics of the examination and the effects of anesthetic drugs may alter the pattern of respiration [19]. Apnea or shallow breathing that is associated with general anesthesia in the absence of endotracheal intubation, as commonly performed in dogs during tracheobronchoscopy [20], may preclude the recognition or the severity of a dynamic AC [3]. In addition, studies comparing the diagnostic utility of I/E-BH CT to tracheobronchoscopy in dogs with an AC are lacking.

This study compared the presence and magnitude of the static and dynamic changes in the intrathoracic airway caliber in dogs with AC, non-collapsible inflammatory airway disease (NCAD) and other non-lower airway respiratory diseases (NLARD) using I/E-BH CT scans and tracheobronchoscopy. The NCAD group included dogs with chronic bronchitis, eosinophilic bronchitis, infectious bronchitis and bronchiectasis. The NLARD group was comprised of respiratory diseases such as neoplasia, suspected crytogenic organizing pneumonia, aerodigestive disorders, bacterial/foreign body/aspiration/interstitial pneumonia and bronchiolar disorders. The objectives were: (1) to describe the differences in airway caliber metrics using I/E-BH CT in dogs with AC, NCAD and NLARD and (2) to compare these metrics for the CT and tracheobronchoscopy scoring in the same three groups. We hypothesized that using I/E-BH CT, the dogs with AC would have larger dynamic variation of their airway caliber and a decreased circularity than the dogs with NCAD and NLARD. Furthermore, we postulated that there would be only a moderate to fair correlation between the paired I/E-BH CT scans and tracheobronchoscopy in the assessment of the AC.

## 2. Materials and Methods

### 2.1. Animals

This retrospective study used the medical record database, University of Missouri Veterinary Health Center, to search from 1 January 2014–30 September 2016 for dogs that underwent I/E-BH CT scans and tracheobronchoscopy. At this institution, any dog requiring a thoracic CT examination as part of the diagnostic workup by the Small Animal Internal Medicine Service underwent a paired I/E-BH CT scan with assisted ventilation to capture the dynamic airway collapse and air trapping that may otherwise be missed on the single-phase inspiratory CT scans [21,22]. The information retrieved included their breed, sex, age, body weight, clinical signs, physical examination findings, the results from airway lavage analysis and the final clinical diagnoses. The final clinical diagnoses encompassed an interpretation of the signalment, history, physical examination and respiratory diagnostics. The dogs with a clinical diagnosis of tracheal collapse, MSB collapse and bronchomalacia were assigned to the AC group. The identification of a lobar bronchial collapse did not necessitate their inclusion in the AC group because in none of the dogs was it the sole or the most severe respiratory abnormality that was identified. When they were available, the number of affected lobar bronchi and the highest severity score were recorded in all of the dogs. The dogs were assigned to the AC group even if they had another comorbid respiratory disease belonging to the NCAD or NLARD groups. The dogs without AC with tracheobronchitis, chronic bronchitis, eosinophilic bronchitis or bronchiectasis comprised the NCAD group. The dogs with respiratory disorders that were not associated with the large airways comprised the NLARD group.

### 2.2. CT Imaging Protocol

The dogs were kept in sternal recumbency until the anesthetic induction in the room housing the CT scanner (64-detector row Aquilion, Canon Medical Systems, Irvine, CA, USA) to prevent dependent atelectasis. The dogs were induced with propofol (Diprivan^®^, Fresenius Kabi USA, LLC, Lake Zurich, IL, USA) that was titrated to ensure its effect. Following the intubation, the dogs were connected to a ventilator (Engstrom Carestation, GE Healthcare, Fairfield, CT, USA) in the volume control mode for the acquisition of the I/E-BH scans. The dogs received positive pressure ventilation using the following initial settings: a tidal volume of 10 mL/kg, 0.4 fraction inspired oxygen (FiO_2_), a respiratory rate set at 10 breaths per minute, an inspiratory:expiratory ratio of 1:3, and a positive end expiratory pressure of 5 cm H_2_O [18,23,24]. The anesthesia was maintained with a constant rate of infusion of propofol at 0.2–0.4 mg/kg/min, which was given to ensure its effect. There were multiple times during the mechanical ventilation where the leak checks were performed. (1) Prior to placing each patient on the ventilator, a system check was performed including a leak check. In the rare instance where the ventilator failed the leak check, the conditions were modified (e.g., the replacement of the anesthetic hoses, the tightening of all of the connections), and the system check was performed again. All of the dogs in the study passed the leak check. (2) In a breath-by-breath fashion, the leak percentage was displayed in real time. If the leak percentage was not zero, the conditions were modified (e.g., addition of more air to the endotracheal tube cuff) until the reading was zero. (3) The ventilator compared the inspiratory and expiratory tidal volumes to detect the leaks and to warn the user of the leaks in the patient circuit. No leak alarms were generated during any of the breath holds in this study. (4) During the acquisition of the breath holds, the ventilator generated a pressure graph during the inspiratory and expiratory holds that were visually observed for a steady plateau that was represented by a horizontal line. In the exceedingly rare instance that the breath was not held at a steady plateau (e.g., if the dog was light under anesthesia), the conditions were modified (e.g., more anesthetic drugs were administered) and the CT series was repeated. 

The timing between the CT acquisition and ventilator-assisted breath holds was accomplished by the direct visualization of the pressure graph that was displayed on the critical care ventilator monitor. The inspiratory images were acquired during the plateau phase of inspiration, mimicking the full(end)-inspiratory phase of the respiration captured in infants and children. The expiratory images were acquired during a short breath-hold pause (5–12 s) that was induced at the end of the expiration. The contiguous transverse CT images were obtained with the dog in sternal recumbency using 64 detector elements × 0.5 mm every rotation, in a 512 × 512 matrix, with a small focal spot size, a 10–30 cm field of view, a pitch of 1.0, 120 kVp, and 250 mAs. Thin-sliced images (0.5–2 mm) which were reconstructed using a high spatial frequency algorithm were transferred to an Empiric PACS system (Encompass, Fujifilm Medical Systems, Lexington, MA, USA). 

The tracheobronchoscopy was performed under general anesthesia according to the canine tracheobronchial roadmap [25]. A red rubber tube terminating in the distal trachea supplied the oxygen, while the endoscope was passed in parallel without use of an endotracheal tube, thus allowing a full assessment of the entire tracheobronchial tree to be performed. The bronchoalveolar lavage was performed immediately after the tracheobronchoscopy examination. 

### 2.3. CT Metrics of AC 

**Figure 1:**
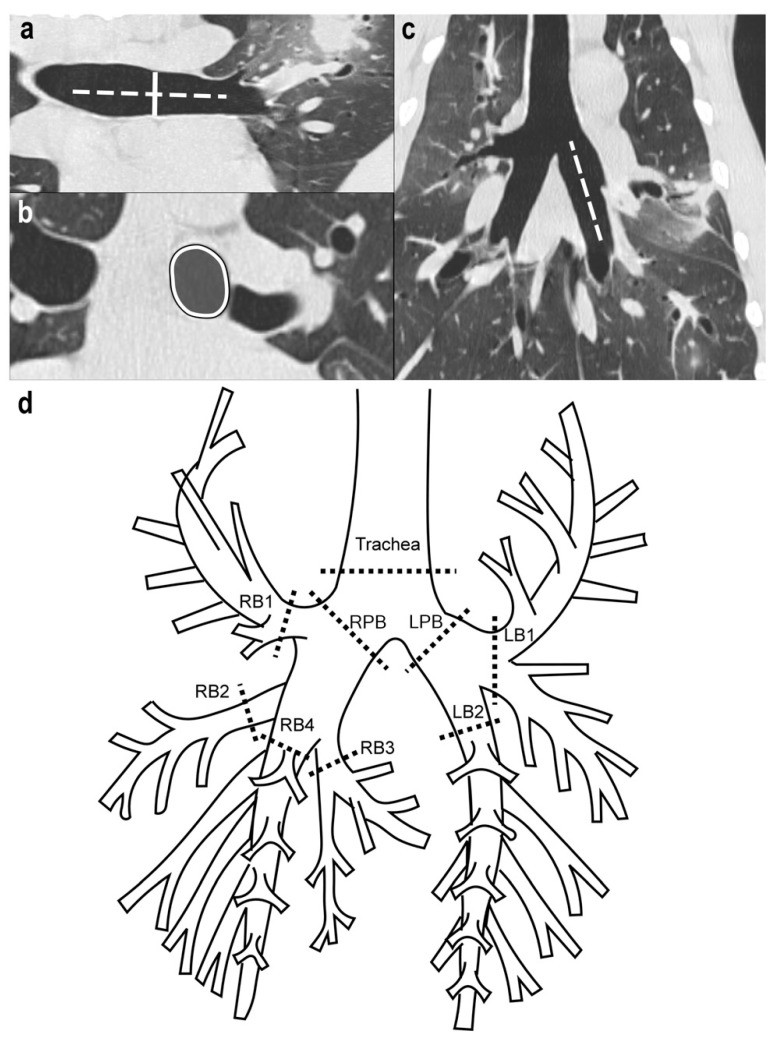
Assessment of diameter- and cross-sectional area (CSA)-derived airway caliber on computed tomography (CT) images. (**a**–**c**) Three-dimensional curved multiplanar reconstruction allowed to us to determine the long axis of each airway based on sagittal (**a**) and dorsal (**c**) planes. As illustrated for LB2, a solid line (**a**) was then drawn perpendicular (dotted line) to the long axis of the airway representing the internal dorsoventral diameter ((**a**), solid line), and the airway is outlined ((**b**), solid line with gray center), thus generating the CSA of the airway. As shown in (**d**), dorsoventral diameters and CSA were obtained for each airway at each location (dotted lines) on the schematic representation of the tracheobronchial tree of the dog on both CT scans and tracheobronchoscopy. All of the measurements were performed by a single observer (A.L.) using non-contrast MPR images with a default lung window (window level = −550 Hounsfield units (HU), window width = 1600 HU), and image magnification of 1000% after importation of CT DICOM images from the UMVHC PACS to a PACS belonging to the Centre Hospitalier Universitaire Vétérinaire, and these were viewed on a dedicated workstation (Impax 6.0, Agfa, Toronto, ON, Canada). RPB, right principal bronchus; LPB, left principal bronchus; RB1, right cranial lobar bronchus; RB2, right middle lobar bronchus; RB3, right accessory lobar bronchus; RB4, right caudal lobar bronchus; LB1, left cranial lobar bronchus; LB2, left caudal lobar bronchus.

Multiplanar reformatting (MPR) was used to obtain images that were oriented perpendicular to the long axis of each airway (Figure 1). The airway caliber measurements from the I/E-BH CTs were performed by a single observer (A. L.) in a standardized fashion with special attention being paid to measuring each airway at a similar location between the dogs and between the phases of respiration. Screen captures allowed the observer to compare the anatomical landmarks between the inspiratory and expiratory CTs and the performances of the measurements at the same level for each airway. The trachea was measured approximately 1 cm cranial to the bifurcation. The left (LPB) and right (RPB) principal bronchi were measured immediately caudal to the bifurcation and cranial to the emergence of the lobar bronchi. The lobar bronchi were measured at their origin and cranial to the emergence of segmental bronchi, as denoted in Figure 1. The measurements included the internal dorsoventral diameter and the cross-sectional area (CSA) of the trachea, RPB, LPB, right cranial lobar bronchus (RB1), right middle lobar bronchus (RB2), right accessory lobar bronchus (RB3), right caudal lobar bronchus (RB4), left cranial lobar bronchus (LB1) and left caudal lobar bronchus (LB2) [25,26]. For the determination of a dynamic airway collapse, the percent airway narrowing (%AN) was calculated by measuring the plateau-inspiratory and end-expiratory airway calibers and using the following equation: ([values]_I_ − [values]_E_)/([values]_I_ × 100, where the values refer to the measurement (diameter or CSA), and “E” and “I” refer to the expiratory or inspiratory series, respectively. To compare the CT measurements and the tracheobronchoscopy grades of AC, the CT-determined %AN based on the diameter and CSA, respectively, was used to assign a score of 0–4 (Table 1).

Static airway collapse was assessed quantitatively by determining the airway circularity from the trachea to the lobar bronchi on the CT images that were acquired during the plateau phase of inspiration at the same level as other measurements. The airway luminal area and circumference were measured using an open-source image processing program (Image J, vs.1.52, NIH, Bethesda, MA, USA), with the circularity being calculated according to the following equation [9]: circularity = (4 × π × area)/circumference^2^. The circularity values range from 0 (severely collapsed airway of flattened or deformed shape in inspiration) to 1 (airway of perfectly or almost perfectly round shape). Static collapse was defined by a low circularity value and a low %AN, translating a deformed airway in inspiration with few changes being observed during expiration. Dynamic collapse, which can be observed in dogs with a both high and a low circularity value, was defined herein by a high %AN.

A reduction in the airway calibers of the segmental and subsegmental bronchi (i.e., bronchomalacia) was scored using the CT scans as follows, Grade 1: a subtle flattening with no peribronchovascular opacification (PBVO; Grade 2: a distorted circular appearance or >50% narrowing with mild to moderate PBVO; Grade 3: a near disappearance of airway lumen with marked PBVO [18]. These three grades were compared to the tracheobronchoscopy grading of bronchomalacia (see below).

### 2.4. Tracheobronchoscopic Grading

The available endoscopic video clips with (*n* = 17) or without (*n* = 22) the reports were reviewed by a board-certified internist (C.R.) who was unaware of the interpretation that had been made by the attending clinician. The scorings for the trachea, mainstem bronchi and lobar bronchi are in Table 1. Segmental and subsegmental bronchial narrowing (i.e., bronchomalacia) were scored as follows [1]: <25%, score = 0; 25–50%, score = 1; 51–75%, score = 2; >75%, score = 3. The airways were examined during spontaneous respiration, and the highest score (on expiration for dynamic collapse) was recorded. When a collapse was documented, the static (no change in grade with respiration) or dynamic (higher grade on expiration) appearance of it was noted. The dogs without video clips or standardized reports (*n* = 12) were excluded from the comparison between the CT and tracheobronchoscopy scores, but they were included in the analysis to assess the first hypothesis. These dogs had limited summaries in their medical record of the salient findings of the tracheobronchoscopy confirming the presence of an AC, but they were without the quantitative documentation of the magnitude of the AC according to the scores that are in Table 1 and described above.

### 2.5. Statistical Analyses 

The statistical analyses were performed by a biostatistician (G.B., see Acknowledgments). A Kruskal–Wallis one-way ANOVA assessed the differences in the age, body weight and airway circularity between the AC, NCAD and NLARD groups. Post hoc testing was performed using Dunn’s pairwise multiple comparison procedure. A Wilcoxon’s signed ranks test assessed the CT variation in the airway calibers (diameter and CSA) between the inspiration and expiration states in the dogs from all of the groups, with the %AN being compared to zero for each airway. The influence of age and weight on the %AN was tested using Spearman’s correlation. The influence of sex was tested using Wilcoxon’s rank sum test. Percent AN was compared between groups using a linear model with age and body mass as covariates. Post hoc testing was performed using Tuckey’s test. The influence of the slice thickness of the measurements was evaluated with univariate one-way ANOVA or one-way ANOVA on the ranks depending on whether the normality (Shapiro–Wilk test) and homoscedasticity (Levene test) were met or not. The post hoc testing procedure used Dunn’s test. The agreement between the CT and tracheobronchoscopy grades was calculated for each airway using the weighted kappa coefficient only in the dogs for which endoscopic video clips or standardized reports using AC grading schemes were available. The statistical significance was set at *p* < 0.05.

## 3. Results

### 3.1. Animals

Fifty-one dogs were studied (AC, *n* = 16; NCAD, *n* = 16; NLARD, *n* = 19; Table 2). The dogs in the AC group had concurrent clinical signs of airflow limitation/functional impairment in addition to I:E-BH CT or tracheobronchoscopy evidence of reduced airway calibers. Of the dogs with an AC, 14/16 (88%) of them had ≥1 comorbid cardiac or respiratory (upper airway, lower airway, small airway/bronchiolar, pulmonary vascular, parenchymal) diseases, with many of these also being shared by the dogs in the other two groups. The median age (range) was 9 (8–16) years in the AC group, 10.8 (0.8–12.6) years in the NCAD group, and 9 (1.5–13) years in the NLARD dogs. There was no difference in the age or sex/reproductive status among the groups (*p* > 0.05). Their body mass varied among the groups (*p* = 0.0016). The post hoc testing revealed that the body mass in the AC group (median (range): 6.7 (3.6–12.3) kilograms (kg)) dogs was smaller than that of the NCAD (17.5 (2.8–60) kg) and NLARD (24.9 (5.8–59.8) kg) dogs. 

### 3.2. Dynamic Airway Wall Narrowing from I/E-BH CT Scans

The %AN was significantly different from zero, indicating the narrowing of all of the airways from the trachea to lobar bronchi at the expiration stage (*p* < 0.0001 for both diameter and CSA). Age and sex did not significantly influence the %AN between inspiration and expiration as calculated from either the diameter or CSA (*p* > 0.08). A significant negative relationship was found between the body mass and %AN of the tracheal diameter between the phases of respiration (*p* = 0.03). No significant relationship between the body mass and %AN was found for the mainstem bronchial or lobar bronchial calibers (*p* > 0.12). The measurements for the LPB, RB1 and LB1 were excluded in two, one and two dogs, respectively, due to the presence of a large neoplastic mass lesion compressing the bronchus or material filling the bronchus.

The %AN using the I/E-BH CT scans was compared among the groups (Table 3). The one-way ANOVA on the ranks found a difference between the groups for the trachea, RPB and RB2 %AN based on the diameter (*p* < 0.001 for the two former, and *p* = 0.01 for RB2) and the CSA (*p* = 0.011, *p* = 0.001 and *p* = 0.004, respectively). The average tracheal and RPB %AN based upon the diameter were greater in the AC group than they were in the NCAD (*p* < 0.007) or the NLARD (*p* < 0.05) groups, and it was greater than in the NCAD group (*p* = 0.007) for the RB2 %AN. There were no significant differences in these measurements between the NCAD and NLARD groups. The %AN for the trachea, RPB and RB2 which were calculated from the CSA was greater in the dogs with AC versus the NCAD (*p* < 0.02) and NLARD (*p* < 0.05) groups. No significant differences between the groups were found in the average %AN (based on diameter or CSA) for all of the remaining airways (*p* > 0.05). Out of fifty-one dogs, seven of them had images which were reconstructed at a 0.05 mm slice thickness which were divided into the NCAD (*n* = 4) and NLARD (*n* = 3) groups. Five and thirty-nine dogs had CT images which were reconstructed at 1 mm (AC, *n* = 1; NCAD, *n* = 2; NLARD, *n* = 2) and 2 mm (AC, *n* = 15; NCAD, *n* = 10; NLARD, *n* = 14) slice thicknesses, respectively. The slice thickness only impacted the measures of the tracheal diameter between 0.5 and 2 mm (*p* = 0.03), while no other measurement was influenced by the selected slice thickness during the CT image reconstruction.

The average circularity of the airways at inspiration was close to one, reflecting that there was minimal flattening (Table 4). The mean tracheal circularity of the AC dogs with significantly smaller than they were in the NCAD (*p* = 0.04) or NLARD (*p* = 0.02) dogs, with no significant difference between the two latter groups. There was no difference in the circularity between the principal and lobar bronchi between the three groups. 

In 39 dogs for which the tracheobronchoscopy score of bronchomalacia was available, 18 dogs lacked I/E-BH CT evidence of the segmental/subsegmental airway caliber reduction. Seven dogs had a score = one, eleven dogs had a score = two and three dogs had a score = three. 

### 3.3. Comparison of CT to Tracheobronchoscopy Score of AC 

The endoscopic video or reports are available in 39 dogs to compare the CT and tracheobronchoscopy data (Table 5, Table 6 and Table 7). An airway caliber reduction of ≥25% was appreciated in 35/39 dogs at, at least one site (trachea, mainstem bronchi, lobar bronchi or segmental/subsegmental bronchi), with 31/35 dogs having a lobar bronchial collapse (with or without other types of AC; Table 2). Based on the tracheobronchoscopy evaluation, the collapse was dynamic (*n* = 19), static (*n* = 12), dynamic and static (*n* = 4) or absent (*n* = 4).

**Figure 2:**
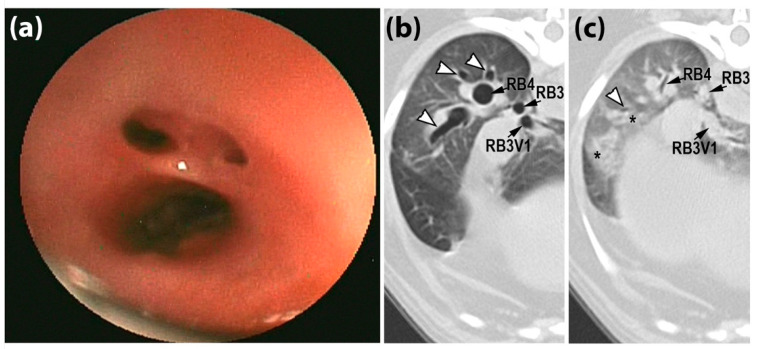
Tracheobronchoscopic and paired inspiratory/expiratory breath-hold CT images from an 8-year-old female spayed Pug in the airway collapse (AC) group. (**a**) The endoscopic image of subsegmental bronchi in the right caudal lung lobe was given a score of 2, reflecting 51–75% luminal diameter narrowing. The paired inspiratory (**b**) and expiratory (**c**) CT images were given a score of 3 as there was near complete disappearance of the lumen of lobar (RB3, RB4), and segmental (RB3V1) and subsegmental (white arrowheads) airways with a marked increase in peribronchovascular opacification on the expiratory series (*). RB3, right accessory lobar bronchus; RB4, right caudal lobar bronchus; RB3V1, first ventral branch of right accessory lobar bronchus.

The CT scoring (median (IQR)) was two (0.75, 2) for the dogs with an AC, zero (0, 1.75) for the dogs with NCAD, and zero (0, 1) for the dogs with NLARD. Bronchoscopic scoring (median (IQR) was two (1, 3) for the AC group, 0.5 (0, 1) for the NCAD group and one (0, 1.5) for the NLARD group. The agreement between the CT and tracheobronchoscopy scores for the trachea, mainstem bronchi and lobar bronchi was achieved in 67/332 of the airways. In 191/323 of the airways, the CT score was higher, and in 74/332 of airways, the CT score was lower than the tracheobronchoscopy score. The weighted kappa coefficients for each airway are detailed in Table 8. The agreement between the CT and tracheobronchoscopy scores for the segmental/subsegmental bronchi (weighted κ = 0.52) was achieved in 18/38 of the dogs, with 5/38 of the dogs having a higher score, and 15/38 of the dogs having a lower score than they did in the tracheobronchoscopy analysis. An example of the tracheobronchoscopy scoring being lower than CT scoring is shown in Figure 2.

## 4. Discussion

The thoracic I/E-BH CT assessed the static and dynamic changes in the airway caliber in the dogs with AC, NCAD and NLARD. In all of the groups, the CT metrics that were derived from the airway diameter and CSA showed a variation in the airway caliber between the phases of respiration as evidenced by all of the airways generating a percentage of airway narrowing at the expiration stage which was significantly different from zero. The dynamic airway wall narrowing was not significantly affected by age or sex, but the dogs of a lower weight (smaller dog breeds) had increased dynamic changes in their tracheal caliber versus the dogs of a higher weight (medium- and large-sized dog breeds). This pattern did not extend to the mainstem or lobar bronchial calibers, possibly suggesting different etiologies or comorbid conditions for the different types of ACs in the small breed dogs. Significant differences in the %AN for the trachea, RPB and RB2 based on the diameter and CSA were found in the AC group versus the NCAD and NLARD groups or for the NCAD group only (i.e., for RB2 %AN based upon diameter). As expected, the dogs with an AC had the highest values of %AN based upon the diameter or on the CSA (Table 3). Except for the trachea, the circularity was not significantly different between the dogs with an AC when it was compared to the NCAD and NLARD groups. When comparing the CT scores to the tracheobronchoscopy scores, the latter considered the criteria standard for the assessment of an AC, only 20% of the airways (from trachea to lobar bronchi) had an agreement between the CT and tracheobronchoscopy scores. The agreement (47%) was higher for the assessment of bronchomalacia. 

Consistent with our hypothesis, the I/E-BH CT scans detected greater changes in the tracheal, RPB and RB2 diameters and CSA in the dogs with an AC versus the NCAD and NLARD groups or the AC versus the NCAD groups only (for RB2 diameter); however, this pattern did not hold true for the remaining airways (Table 3). The lack of differences between the %AN of the lobar bronchi is likely because all three groups had dogs with lobar bronchial collapses which were detected with the tracheobronchoscopy. A lobar bronchial collapse is unique from other types of ACs in that (1) it is not a discrete clinical syndrome, and (2) it may affect a single or very few lobar bronchi with minimal impact on global respiration function [29]. In our study, a lobar bronchial collapse always occurred in the presence of another respiratory disorder. For these reasons, we chose to not include the dogs with just this abnormality in the AC group. A lobar bronchial collapse is a common subjective finding during tracheobronchoscopy especially in certain breeds [5], with clinical signs that are generally overshadowed by comorbid conditions. This was consistent with our study in which the CT metrics of the decreased airway caliber of the lobar bronchi at the expiration stage were seen in all three groups of dogs with no significant differences in the %AN based upon the diameter or CSA except for RB2 based on the diameter and CSA in the AC dogs. Using tracheobronchoscopy, 31/39 dogs had evidence of at least one lobar bronchus, displaying a narrowing of ≥25%. This underscores that a bronchial collapse is an abnormality which should be recognized, documented, and monitored, but additional studies will be needed to correlate a lobar bronchial collapse with specific clinical signs and function impairment. 

Among all of the measurements, only the tracheal diameter was influenced by the slice thickness. Since the slice thickness impacts the image through longitudinal partial volume effects, the use of a slice thickness of 75% of the size of the object of interest has been recommended [30]. Most of the dogs (except one) in our study had tracheal luminal diameters that were greater than 5 mm and thus, a size above this is the minimum recommended threshold.

The agreement between the CT and tracheobronchoscopy scores in the dogs were not as close as they were in people [14,31]. In people, an AC is simply defined by a >50% reduction in the airway lumen during expiration in CT and tracheobronchoscopy testing [31,32,33], corresponding to the current study’s scores of three or four from the trachea to lobar bronchi and two or three for the segmental/subsegmental airways. Although the current study employed scores to capture smaller degrees of ACs, this species discrepancy cannot be fully explained. Using a definition of >50% AC for both the tracheobronchoscopy and CT and using tracheobronchoscopy as the criterion standard, 7/18 tracheas/principal bronchi (Table 5) and 8/35 lobar bronchi (Table 6) followed this definition. The best agreement was for bronchomalacia, with 14 dogs having tracheobronchoscopy scores with a >50% collapse and of those, 11 dogs had CT scores of >50% (Table 7).

A routine CT scan, performed with just end-inspiratory imaging, underdiagnoses ACs in people [16]. The dynamic CT was developed to detect ≥50% of the collapsibility of the airways [34]. Awake functional bronchoscopy in people confirms the results of the dynamic CT during quiet breathing [35], but it cannot be performed in dogs. The differences in the agreement between the CT and tracheobronchoscopy in the dogs compared to humans may in part be explained by them undergoing anesthetized versus awake and spontaneous breathing versus assisted ventilation examinations. However, in infants and young children, one study showed a high agreement between the CT and tracheobronchoscopy scores that were obtained under anesthesia with positive-pressure ventilation and spontaneous breathing, respectively [14], although the high severity of the condition in these patients may have contributed to a good agreement. In the dogs, the CT tended to assign higher scores than the tracheobronchoscopy did for milder ACs of grades 1–2. Indeed, for the trachea and mainstem bronchi, the CT identified 10–50% of the collapses in 98 airways and the tracheobronchoscopy identified 10–50% of the collapses in 18 airways (Table 5). For the lobar bronchi, the CT identified 10–50% of the collapses in 170 airways and the tracheobronchoscopy identified 10–50% of the collapses in 78 airways (Table 6). Compared to the tracheobronchoscopy, the CT detected more airways with collapses (i.e., grades 1–4); Table 5 and Table 6). 

There are other reasons for the poor agreement between the imaging modalities in the assessment of AC. Although MPR was used to obtain perpendicular sections of each airway based on their long axis, thus minimizing the measurement under- or overestimation due to inappropriate orientation, this approach is not perfect if the bronchi do not follow a linear path (e.g., as for RB2, RB1 and LB1). The impact of the patient size on the field of view and the subsequent voxel size may also have played a role. Toy/small breed dogs have smaller airways when they are compared to large breed dogs; a small change in the caliber may have a greater impact on the percentage calculations. In contrast, the tracheobronchoscopy scoring is subjective with the airway dimension being tailored to screen size, thus dampening the effect of the patient size. Additionally, the airways remaining circular in shape during the tracheobronchoscopy despite there being a circumferential decrease in the diameter would be scored as “0” in contrast to the CT where it is more likely to be scored as “1” or higher. This study highlights the difficulty of comparing the modalities whose inherent properties make it difficult to apply identical criteria during their evaluation. The application of a scoring system is not necessarily synonymous with the clinical/functional grades of the collapse. Establishing cut-offs for classifying the dogs as having AC based only on the I/E-BH CT is challenging. Applying a 50% collapsibility cut-off for diagnosing a lobar collapse does not appear to do justice as many dogs with a collapse according to the tracheobronchoscopy have low CT scores, whereas the dogs without clinical signs of AC can receive scores of three [11].

A study limitation is the retrospective nature as it relies on review of video clips or reports of the tracheobronchoscopy findings, leading to the exclusion of dogs with incomplete or lost data. Enrolling dogs with more costly I/E-BH CT scans (versus standard CT acquisition) may also have introduced a bias. The limited number of dogs in each group may also have contributed to the fair agreements that were found between the I/E-BH CT and tracheobronchoscopy scores.

## 5. Conclusions

Objective measurements, images that are captured during full inspiration and expiration, and the ability to evaluate the sequelae of segmental/subsegmental ACs (i.e., bronchomalacia) on the downstream pulmonary parenchyma represent important advantages of I/E-BH CT. A single scoring system that was applied to two different modalities, I/E-BH CT and tracheobronchoscopy, did not provide a high agreement for the assessment of ACs in dogs with spontaneous respiratory disease. Future studies correlating magnitude of ACs with functional impairment are needed. 

## Figures and Tables

**Table 1:** Score assignment (0–4) for both inspiratory and expiratory breath-hold computed tomographic (CT) measurement and tracheobronchoscopic grades of airway collapse to facilitate comparison between these two imaging modalities. The scoring system was based on a modification of Tangner’s original tracheal collapse scores [27].

Dynamic Airway Narrowing Magnitude ^1^	Score ^2^
<10%	0
10–25%	1
26–50%	2
51–75%	3
>75%	4

^1^ This scoring system was applied to the trachea, mainstem bronchi and lobar bronchi at similar locations using CT and tracheobronchoscopy. The CT-determined percent airway narrowing (%AN) based upon diameter and cross-sectional area, respectively, was used for scoring; ^2^ Whenever complete airway collapse on expiration prevented CT measurements, a score of 4 was assigned.

**Table 2:** Study population demographics and clinical diagnoses according to group including dogs with airway collapse (AC), dogs with non-collapsible airway disease (NCAD) and dogs with other non-lower airway respiratory diseases (NLARD).

Dog	Age (years)	Sex	Weight (kg)	Breed	Clinical Diagnosis	Lobar Bronchial Collapse (Number of Affected Bronchi, Highest Score) ^1^
Airway Collapse Group (AC)						
1	12.2	FS	12.3	American Cocker Spaniel	Presumptive pulmonary carcinoma, BE, BM, CB	NA
2	7.8	MC	8.6	Mixed Breed	TC, MSBC, CB, bronchiolitis, BE	5, 4
3	10	MC	8.3	Brussels Griffon	TC, MSBC, CB, BE	6, 3
4	8.7	MC	8.1	Brussels Griffon	Dynamic upper airway obstruction (elongated soft palate), TC, BM	5, 3
5	12.6	MC	3.6	Chihuahua	TC, MSBC, BM, MVDD, PH, pulmonary fibrosis ^2^	4 (NA = 2), 4
6	16	FS	4.5	Chihuahua	TC, MSBC, BE, BM, PTE ^2^	6, 2
7	11.5	FS	5.8	Chinese Crested	CB, BE, TC, constrictive bronchiolitis obliterans with pulmonary fibrosis ^2^	5, 3
8	13.4	MC	6.5	Dachshund	CB, BE, BM, suspect aspiration pneumonia	1, 3
9	11	MC	6.0	Dachshund	TC, MSBC, EB, BE, BM, MVDD	2, 4
10	8.3	MC	11.7	French Bulldog	Aerodigestive disease (megaesophagus, gastroesophageal reflux, and aspiration pneumonia), TC, MSBC, BM	6, 4
11	12.2	FS	5.8	Jack Russel Terrier	TC, MSBC, BE, BM, paratracheal and paraesophageal chronic inflammation/steatitis with fibrosis^2^	3 (NA = 2), 3
12	8	FS	5.1	Maltese	TC, CB	1, 1
13	13.1	FS	9.1	Pekingese	Dynamic pharyngeal collapse, PH, CB, MVDD, TC, MSBC, BM	6, 4
14	10.6	MC	4.3	Miniature Poodle	TC, MSBC, BM	6, 4
15	8	MC	6.9	Miniature Poodle	TC, MSBC, BM	6, 4
16	8	FS	7.9	Pug	TC, BE, BM, bronchiolar disease, pulmonary fibrosis, acute lung injury ^2^	4 (NA = 2), 3
Non-collapsible Inflammatory Airway Disease Group (NCAD)						
17	2	MC	14.5	Basenji	EB, BE	NA
18	0.8	FS	22.4	Boxer	CB	0
19	12.3	MC	2.8	Chihuahua	BE, bronchiolar disease	5, 2
20	10.1	MC	6.2	Dachshund	Pneumocystis bronchopneumonia, BE, CB	4, 4
21	13	FS	7.0	Dachshund, Longhaired Standard	CB, BE, pulmonary fibrosis^2^	5, 2
22	10	FS	20.5	German Shorthaired Pointer	CB, subtle small nodules on CT (etiology undetermined)	3, 2
23	1.5	MC	60	Great Pyrenees	Suspect canine hyperreactive airway disease (atelectasis R middle lung lobe, hyperinflation, air trapping, AW wall thickening)	2, 1
24	11.6	MC	27.3	Hovawart	Laryngeal paralysis, BE	0
25	11	M	35.5	Labrador Retriever	Bilateral laryngeal paralysis, CB	4, 2
26	2.5	FS	42.4	Labrador Retriever	Presumptive canine infectious respiratory disease complex (infectious tracheobronchitis)	NA
27	4	FS	22.5	Mixed breed	Foreign body pneumonia, BE	0
28	1.3	FS	20.8	Pointer	Recurrent bacterial pneumonia, BE, CB	NA
29	7	FS	7.6	Shetland Sheepdog	EB, BE	NA
30	9	FS	8.1	Shih Tzu	CB, BE	1, 2
31	9	FS	14.2	Welsh Corgi, Pembroke	Lymphoplasmacytic rhinitis^2^, CB	2, 1
32	12.6	FS	7.8	West Highland White Terrier	CB, suspect pulmonary fibrosis	NA
Non-Lower Airway Respiratory Diseases Group (NLARD)						
33	2	M	19.0	Basset Hound	Suspect cryptogenic organizing pneumonia (steroid-responsive)	0
34	9	FS	29.4	Bernese Mountain Dog	Pulmonary adenocarcinoma	Compression by mass
35	12	FS	18.2	Border Collie	Bilateral laryngeal paralysis, aerodigestive disease	4, 4
36	9.1	MC	24.4	Brittany Spaniel	Mineralized osteomas, epiglottic retroversion	2, 1
37	12.5	MC	5.8	Dachshund, Miniature	Suspect cryptogenic organizing pneumonia (steroid-responsive)	3, 2
38	13	FS	6.9	Dachshund	Chronic lung congestion, suppurative interstitial pneumonia ^2^	4, 2
39	7	MC	10.5	Fox Terrier, Smooth	Foreign body pneumonia	NA
40	1.5	FS	10.7	German Shepherd Dog	Bacterial pneumonia, pyothorax	NA
41	3	MC	26.4	German Shepherd Dog	Structural disease of nose and nasopharynx, constrictive bronchiolitis obliterans ^2^	NA
42	10	FS	59.8	German Shepherd Dog	No cause found for panting, morbid obesity?	NA
43	1.5	FS	24.9	Giant Schnauzer	Streptococcus canis bronchopneumonia/necrotizing bronchiolitis ^2^	2, 1
44	9.6	F	37.5	Labrador Retriever	Histiocytic sarcoma	2, 1
45	6.1	FS	26.8	Mixed Breed	Emphysema ^2^	0
46	9.8	MC	40.9	Mixed Breed	Pulmonary neoplasia	NA
47	3.5	M	31.0	Pointer	Histiocytic sarcoma (pulmonary involvement) ^2^	NA
48	5	M	6.3	Shih Tzu	Pulmonary blastomycosis	NA
49	8	FS	18.7	Siberian Husky	Histiocytic sarcoma (pulmonary involvement) ^2^	4, 4
50	9.5	MC	28.2	Welsh Springer Spaniel	Rhinitis, laryngeal dysfunction, aerodigestive disorder (dysphagia, aspiration pneumonia)	0
51	10.3	FS	37.2	Weimaraner	Pulmonary papillary adenocarcinoma	2, 1

^1^ Number of affected lobar bronchi (amongst RB1, RB2, RB3, RB4, LB1 and LB2), highest score obtained on tracheobronchoscopy. Dogs for which tracheobronchoscopy was either not performed or performed, but who are missing video clips of lobar bronchi or lacking information related to assessment of lobar bronchi in the report, were recorded as not available (NA); ^2^ Diagnosis obtained via gross examination at necropsy or via histopathology (antemortem biopsy or necropsy). Airway, AW; Bronchiectasis, BE; Bronchomalacia, BM; Chronic bronchitis, CB; Eosinophilic bronchitis, EB; Mainstem bronchial collapse, MSBC; Mitral valve degenerative disease, MVDD; Not available, NA; Pulmonary hypertension, PH; Pulmonary thromboembolism, PTE; Right, R; Tracheal collapse, TC.

**Table 3:** Percentage of dynamic airway narrowing (median (interquartile range (IQR))) in dogs with airway collapse (AC), with non-collapsible inflammatory airway disease (NCAD) and non-lower airway respiratory disease (NLARD) based on diameter and cross-sectional area measured on paired inspiratory/expiratory breath-hold CT scans.

Site	AC	NCAD	NLARD
Based upon dorsoventral luminal diameter			
Trachea	17.9 (10.1, 45.4) ^1^	7.2 (0.9, 13)	8.1 (3.2, 11.3)
Right Principal Bronchus	24.1 (14.4, 36.6) ^1^	9.3 (1.6, 14.9)	9 (4.1, 16.9)
Left Principal Bronchus	17.3 (5.3, 49.7)	12.6 (7.6, 24)	14.2 (3.7, 17.9)
Right cranial lobar RB1	43.5 (5.5, 58.5)	16.2 (1.1, 26.1)	16.4 (10.9, 21)
Right middle lobar RB2	37.2 (6.6, 52) ^2^	2.2 (0, 22.3)	13.3 (6.5, 27.3)
Right accessory lobar RB3	14.9 (0.6, 27.8)	11.3 (0, 17.2)	14.3 (7.1, 25)
Right caudal lobar RB4	24.1 (0.3, 33.5)	9.1 (0, 18.7)	16.4 (5.4, 18.9)
Left cranial lobar LB1	28.1 (11.3, 38.2)	12.3 (2.7, 32.7)	12.0 (4.5, 22.2)
Left caudal lobar LB2	22.5 (5.5, 36.9)	12.6 (6.4, 24.9)	15.8 (6.4, 24.9)
Based upon cross-sectional area			
Trachea	16.2 (9.8, 36) ^1^	7.6 (1.8, 14)	4.8 (-0.2, 15.6)
Right Principal Bronchus	35.2 (21.6, 57.3) ^1^	9.9 (4.6, 18.6)	14.9 (6.1, 24.7)
Left Principal Bronchus	33.5 (7.6, 32.4)	16.9 (7.6, 32.4)	17.4 (9.4, 30.3)
Right cranial lobar RB1	52 (9.7, 75.4)	24.5 (8.8, 43.1)	26.7 (16.1, 38.6)
Right middle lobar RB2	58.8 (33.1, 78.4) ^1^	27 (6, 39.1)	33 (8.3, 43.4)
Right accessory lobar RB3	29.4 (20.7, 33)	20.6 (5.1, 34.0)	23.6 (11.2, 36.7)
Right caudal lobar RB4	46.2 (6.8, 51.3)	18.6 (1.7, 31.5)	26.3 (12.9, 32.4)
Left cranial lobar LB1	31.9 (11, 55.5)	24.9 (15.7, 45.6)	29.6 (12.7, 43)
Left caudal lobar LB2	27.8 (14, 58.3)	27.7 (8.7, 39.9)	26.4 (8.5, 40.2)

Post hoc testing results (Dunn’s Method) showed significant differences (*p* <0.05) between AC and both ^1^ NCAD and NLARD or between AC and ^2^ NCAD only.

**Table 4:** Airway circularity values (median (IQR)) in dogs with airway collapse (AC), with non-collapsible inflammatory airway disease (NCAD) and non-lower airway respiratory disease (NLARD).

Site	AC	NCAD	NLARD
Trachea	0.95 (0.90, 0.96) *	0.97 (0.96, 0.98)	0.97 (0.97, 0.98)
Right Principal Bronchus	0.96 (0.93, 0.97)	0.96 (0.95, 0.97)	0.97 (0.96, 0.97)
Left Principal Bronchus	0.94 (0.91, 0.97)	0.96 (0.93, 0.97)	0.97 (0.96, 0.98)
Right cranial lobar RB1	0.96 (0.96, 0.97)	0.96 (0.96, 0.97)	0.96 (0.94, 0.97)
Right middle lobar RB2	0.95 (0.94, 0.97)	0.96 (0.94, 0.97)	0.95 (0.94, 0.96)
Right accessory lobar RB3	0.97 (0.96, 0.97)	0.95 (0.94, 0.97)	0.97 (0.94, 0.97)
Right caudal lobar RB4	0.97 (0.96, 0.97)	0.97 (0.96, 0.97)	0.97 (0.96, 0.98)
Left cranial lobar LB1	0.95 (0.93, 0.96)	0.96 (0.95, 0.97)	0.95 (0.95, 0.96)
Left caudal lobar LB2	0.97 (0.96, 0.97)	0.97 (0.96, 0.97)	0.97 (0.97, 0.98)

* *p* < 0.05.

**Table 5:** Comparisons of scores for airway caliber narrowing using inspiratory/expiratory breath-hold CT and tracheobronchoscopy (tracheobronchoscopy) for the trachea and mainstem bronchi (right and left principal bronchi). The CT-determined percentage of dynamic airway wall narrowing based on dorsoventral diameter and subjective tracheobronchoscopic narrowing were each used to assess a score (in bold) from 0–4 as shown in Table 1. Numbers which are not in bold reflect the number of airways evaluated for each grade of collapse for each imaging modality.

				CT			
	Grade of Collapse	0	1	2	3	4	Total
	**0**	4	61	6	0	0	71
	**1**	2	6	5	0	0	13
**TB**	**2**	0	5	6	2	0	13
	**3**	2	3	1	2	1	9
	**4**	0	3	2	3	1	9
	**Total**	8	78	20	7	2	115

**Table 6:** Comparisons of scores for airway caliber narrowing using inspiratory/expiratory breath-hold CT and bronchoscopy (B) for the six lobar bronchi. The CT-determined percentage of dynamic airway wall narrowing based on dorsoventral diameter and subjective bronchoscopic narrowing were each used to assess a score (in bold) from 0–4 as shown in Table 1. Numbers which are not in bold reflect the number of airways evaluated for each grade of collapse for each imaging modality.

				CT			
	Grade of Collapse	0	1	2	3	4	Total
	**0**	11	54	36	3	0	104
	**1**	8	21	12	2	0	43
**B**	**2**	3	13	11	7	1	35
	**3**	3	6	7	5	1	22
	**4**	1	4	6	2	0	13
	**Total**	26	98	72	19	2	217

**Table 7:** Scoring of airway caliber narrowing of segmental/subsegmental bronchi using inspiratory/expiratory breath-hold CT and bronchoscopic scoring. Reduction in airway calibers of segmental and subsegmental bronchi were scored using bronchoscopy as follows (modified from [6]: 0–25%, score = 0; 26–50%, score = 1; 51–75%, score = 2; >75%, score = 3.

				CT		
	Grade of Collapse	0	1	2	3	Total
	**0**	11	0	0	0	11
	**1**	6	4	3	0	13
**B**	**2**	1	2	2	2	7
	**3**	0	0	6	1	7
	**Total**	18	6	11	3	38

**Table 8:** Weighted kappa coefficients scores and interpretation of magnitude of agreement obtained from comparison of airway collapse between inspiratory/expiratory breath-hold CT and tracheobronchoscopy in dogs with spontaneous respiratory disease based upon CT-derived airway diameter (DVd) and cross-sectional area (CSA) for trachea, mainstem bronchi and lobar bronchi and for bronchomalacia severity scoring systems for segmental and subsegmental bronchi.

	Weighted Kappa Coefficient ^1^	Interpretation ^2^
1. Trachea, mainstem bronchi and lobar bronchi		
Trachea (DVd) ^3^	0.2895	Fair
Trachea (CSA) ^4^	0.2519	Fair
Right Principal bronchus (DVd) ^3^	0.1674	Slight
Right Principal bronchus (CSA) ^4^	0.1502	Slight
Left Principal bronchus (DVd) ^3^	0.2550	Fair
Left Principal bronchus (CSA) ^4^	0.3184	Fair
Right cranial lobar RB1 (DVd) ^3^	0.2977	Fair
Right cranial lobar RB1 (CSA) ^4^	0.1303	Slight
Right middle lobar RB2 (DVd) ^3^	0.2382	Fair
Right middle lobar RB2 (CSA) ^4^	−0.1250	None
Right accessory lobar RB3 (DVd) ^3^	0.1529	Slight
Right accessory lobar RB3 (CSA) ^4^	0.0166	Slight
Right caudal lobar RB4 (DVd) ^3^	0.1866	Slight
Right caudal lobar RB4 (CSA) ^4^	0.0899	Slight
Left cranial lobar LB1 (DVd) ^3^	0.0517	Slight
Left cranial lobar LB1 (CSA) ^4^	−0.0756	None
Left caudal lobar LB2 (DVd) ^3^	0.0565	Slight
Left caudal lobar LB2 (CSA) ^4^	0.0585	Slight
2. Segmental and subsegmental bronchi	0.52	Moderate

^1^ Agreement between inspiratory/expiratory CT and tracheobronchoscopy airway collapse grading was performed for each airway with weight kappa coefficient; ^2^ Interpretation of weight kappa coefficients [28]: poor agreement for values <0; slight agreement for values between 0 and 0.2; fair agreement for values between 0.21–0.4; moderate agreement for values between 0.41 and 0.6; substantial agreement for values between 0.61 and 0.8; almost perfect agreement for values between 0.81 and 1.0; ^3^ Based upon CT metrics derived from dorsoventral airway diameter (DVd); ^4^ based upon CT metrics derived from airway cross-sectional area (CSA).

## Data Availability

The data presented in this study are available on request to the corresponding author.
